# Undiagnosed cancer symptoms in the community: does poor quality of life influence the decision to seek help?

**DOI:** 10.1007/s11136-018-2088-9

**Published:** 2019-01-22

**Authors:** Nicola Gartland, Hannah Long, Suzanne M. Skevington

**Affiliations:** Manchester Centre for Health Psychology and International Hub for Quality of Life Research (IHQoLR), Faculty of Biology, Medicine and Health, University of Manchester, Manchester, MP13 9PL UK

**Keywords:** Cancer, Quality of life, Early diagnosis, Help-seeking, Symptoms

## Abstract

**Purpose:**

Although a cancer diagnosis is linked with decrements to quality of life (QoL), it is unknown exactly when QoL starts to deteriorate, and whether this occurs during the pre-diagnostic pathway. This study aimed to examine QoL during this phase, and in addition investigate whether QoL levels influence decisions about seeking professional help. This is important, because early diagnosis is linked to lower cancer mortality rates.

**Methods:**

Working alongside a Cancer Research UK Roadshow in socially deprived communities, the recent QoL of adult visitors was assessed, *before* attending primary care. Using a cross-sectional design, we compared QoL in those presenting a potential cancer symptom/sign, with others seeking lifestyle advice to reduce cancer risk. Self-reported QoL (WHOQOL-BREF), and intention to seek help, were measured.

**Results:**

Of 107 recruited, 50% were men. The potential cancer symptom group reported significantly poorer general QoL and psychological QoL, than lifestyle controls. Prior poorer physical QoL predicted stronger intentions to consult a general practitioner (GP) in the next 2 weeks, when controlling for symptom presence.

**Conclusions:**

QoL is poorer for those with potential cancer symptoms, before they first seek advice from primary care. Poorer physical QoL is associated with stronger intentions to make a GP appointment. An implication for longer term health is that if public awareness about the impact of symptoms on QoL was raised, this could provide an impetus to seek help.

## Introduction

Early diagnosis of cancer results in a better prognosis than later diagnosis [1, 2], but many complex factors surround the process of obtaining an early diagnosis, and these affect eventual outcomes [3]. As many cancers are diagnosed after reporting signs or symptoms to a GP [4], the timing of help-seeking for cancer symptoms represents a potentially modifiable route to improving early diagnosis rates [2, 5].

The Pathways to Treatment Model highlights help-seeking for bodily changes as an important step towards gaining a diagnosis of cancer [6]. This model contains a descriptive framework of five events with a sequence of dynamic processes through the pathway: detecting bodily changes, perceiving reasons to discuss a symptom with a health care provider (HCP), the first HCP consultation, receiving a diagnosis, and starting treatment. However, the period between symptom detection and first HCP presentation varies widely and contains intervals of both appraisal and of help-seeking. Factors that influence these two intervals include misinterpreted symptoms, self-management, and competing priorities, although these mechanisms are not fully understood [7].

Physical symptoms are associated with impaired quality of life (QoL) because of the functional limitations that these symptoms often impose when the condition becomes chronic [8, 9], but less is known about how *potential* cancer symptoms impact on QoL, especially during the earliest stage of the pathway, before seeking help. Symptom detection usually occurs within the community, so this information is hard to access by researchers, and in clinical practice. Consequently, identifying and recruiting individuals who have noticed a change in their body, but not yet sought help from their GP, is a significant challenge for anyone aiming to study this earliest stage. Without the resources to perform a large-scale population survey to identify such individuals, instead we utilised an opportunity to work alongside the Cancer Research UK North West Roadshow team. This peripatetic unit visits towns in north-west England, aiming to engage ‘hard-to-reach’, socio-economically deprived communities with health messages, and increasing awareness about the symptoms and signs of cancer. Roadshow nurses draw in the public to discuss cancer, and ‘signpost’ to GPs when appropriate. This provides unique and efficient method of identifying and accessing community adults who may have a cancer symptom that they have not discussed with their GP.

Recent research indicated that at the pre-diagnosis stage, QoL is lower in people who are later diagnosed with cancer, compared to a cancer-free comparison group, and it was suggested that this might be because of the impact of symptoms on QoL [10]. It is likely that this effect is multifactorial; in addition to the direct physical effects of experiencing a potential symptom, other dimensions of QoL could also be affected such as sleep loss, greater negative feelings, and weakened social relationships. Furthermore, deteriorating QoL may also motivate or deter a decision, and the timing of that decision, to seek formal help. This is the topic of the present research.

The present research is underpinned by the World Health Organisation’s (WHO) subjective definition of QoL as: ‘An individual’s perception of their position in life, in the context of the culture and value systems in which they live, and in relation to their goals, expectations, standards and concerns’ [11]. This definition influenced the development of the WHOQOL patient-, and person-reported outcome measures (PROMs) for cross-cultural use in a wide range of sick and well populations. Cancer-specific PROMs like the EORTC [12] are widely used to assess QoL in patients receiving cancer diagnosis and treatments [13–15], but generic measures are more appropriate for assessing QoL and health in mixed community samples and can monitor cyclical fluctuations between well and illness states during treatment and recovery [16]. The WHOQOL-BREF used in the present research measures 25 facets of QoL, organised in four QoL ‘domains’: physical, psychological, social, and environmental. While it is likely that physical symptoms will impact on physical QoL in the current context, it is also possible that symptoms will have wide effects on other domains.

Studies of QoL have demonstrated its prognostic value; QoL data can provide unique information beyond that gained from clinical indicators of health; for instance, by improving prognostic accuracy in cancer randomised controlled trials (RCTs) by 5.9–8.3% [17]. The utility of QoL data can therefore extend beyond its conventional role as an outcome measure. Indeed, evidence from a systematic review indicates that QoL levels are associated with help-seeking behaviour for a range of potential cancer symptoms, including pain, urinary, bowel and respiratory symptoms. This review identified 15 studies (*N* = 6646) which assessed the relationship between QoL and help-seeking behaviours in primary care for a range of physical symptoms listed by Cancer Research UK as potential signs or symptoms of cancer. Thirteen studies reported positive evidence of the QoL and help-seeking relationship; the two remaining studies showed limitations which could have introduced bias [18, 19]. Consequently, we conclude that greater detriments to QoL are associated with help-seeking in primary care. This effect was found for a range of potential cancer symptoms, and also across different cultures. Prospective analyses indicated that detriments in QoL *predict* subsequent help-seeking, which suggests QoL as a motivational factor in the decision to seek help from primary care for a potential cancer symptom [20, 21]. The importance of QoL is further supported by the observation that while other indicators of symptom severity and health status were found in some studies to predict help-seeking behaviours, QoL retained predictive power in these regression models, and sometimes outperformed symptom measures [21, 22]. However, there were very few high-quality prospective studies, highlighting the need for more rigorous testing of the causal relationship.

The present research was designed to gather preliminary data to address whether visitors to a Cancer Research UK roadshow with a potential cancer symptom/sign, report poorer QoL in the 2 weeks before the visit. It was expected that lower levels could be associated with a stronger intention to consult a GP in the period immediately following the visit. If confirmed, this would lend support to growing evidence that QoL is among the motivating factors in the decision to seek formal help from primary care.

Two hypotheses were tested:

1. Participants with a potential cancer symptom/sign will report poorer QoL in the 2 weeks prior to the visit than a comparison group seeking lifestyle advice.
2. Poorer QoL before the visit will predict stronger intentions to consult a GP in the 2 weeks following the visit.

## Methods

### Sampling and recruitment

Recruitment was conducted at 43 socio-economically deprived urban sites visited by the Cancer Research UK North West Roadshow over two roadshow seasons (June 2015–September 2016). The roadshow offers body mass index (BMI) and ‘smokerlyzer’ tests, as well as private conversations with qualified nurses. The specialist nurses do not diagnose, but ‘signpost’ visitors to consult their GP when appropriate. The roadshow visits locations in the summer months, typically for three consecutive mid-week days. Most locations were outdoors, in high streets and shopping centres. The field researcher (NG) collected data in a tent adjacent to the Cancer Research UK unit and recruited participants after their visit. Nurses were briefed about study criteria and introduced visitors to the researcher.

### Design

A cross-sectional design was used in the present study. Inclusion criteria were either (i) a bodily change (sign/symptom) that had not been reported to a GP (‘symptom’ group); or (ii) wanting to discuss lifestyle changes to reduce cancer risks (alcohol, smoking, diet, ‘sunsmart’, physical activity, weight control, screening attendance) (‘lifestyle’ group). Exclusion criteria were: (a) a previous consultation with a GP about the current bodily change; (b) a current diagnosis of cancer; and (c) concerns solely about other people (e.g. family, friends). Eligible visitors were informed and invited to participate. Willing participants were screened for suitability: (1) age 18 +; (2) reads and speaks fluent English; (3) no vision difficulties (with contact lenses/glasses). Participants gave fully informed, written consent. Ethical approval was granted by the University Ethics Committee (ref. 15163).

### Procedure

Information was recorded on socio-demographic features (birth date, gender, education, marital and employment status, ethnicity), health status (current illness and type), QoL, and help-seeking behaviour. A laptop computer was used to enter data on site, via the study website hosted on the university server.

### Materials

#### Quality of life (WHOQOL-BREF)

**Fig. 1:**
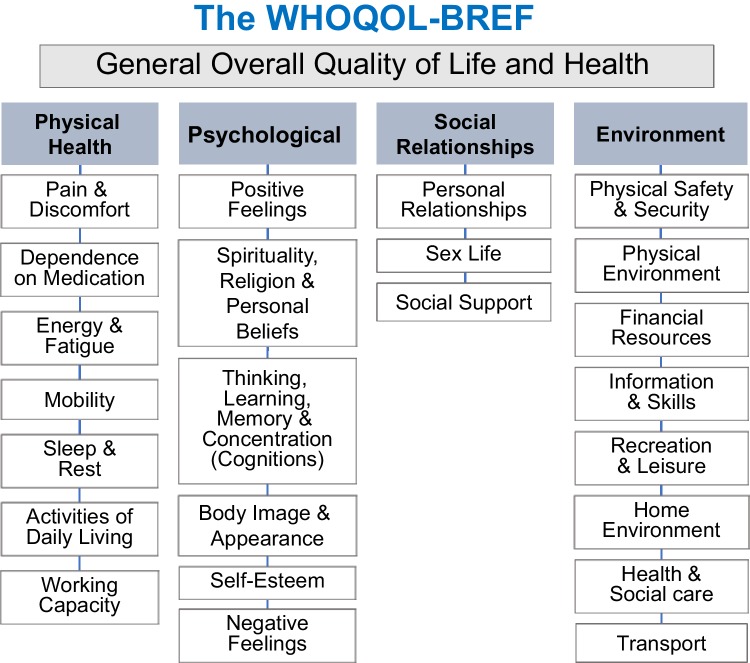
Structure of the WHOQOL-Bref showing the broad domains and the facets within the domains

The WHOQOL-BREF is a 26-item multi-dimensional measure that assesses subjective QoL over the past 2 weeks (5-point Likert interval scales with various question specific response frames e.g. not at all to an extreme amount/very dissatisfied to very satisfied; scores of 1–2 represent poor QoL, 3 average QoL, and 4–5 good to excellent QoL). Two items assess general QoL (overall QoL and general health); the other 24 items each assess one facet of QoL and are scored as four domains: physical, psychological, social and environmental QoL (see Fig. 1). The internationally standardised WHOQOL-BREF shows good psychometric properties [23]. The UK language version is reliable, valid, and sensitive to changes in clinical and social conditions [24].

#### Depression (patient health questionnaire–2(PHQ-2))

As depression can reduce QoL across many dimensions [25], the PHQ-2 was used to assess depressive symptoms. Two items record the frequency of depressed mood and anhedonia in the past 2 weeks, and are rated on a 4-point Likert interval scale, and summed (range 0–6) [26].

#### Strength of intention to seek help

The strength of intention to seek help from a GP in the short-term was measured using a single item: ‘How likely is it that you will go to the GP in the next 2 weeks?’ A 5-point rating scale ranged from ‘very unlikely’ (1) to ‘very likely’ (5), with a mid-point (3).

### Analysis

Using the UK WHOQOL manual (with UK SPSS syntax file), missing data rules were applied. Scored items were transformed for each domain to provide a domain score (0–100; where a higher score represents better QoL and 50 represents average QoL), and the two general items meaned. As the general QoL mean shared variance with all domains, it was analysed separately. Missing data in domain scores (*n* = 1) were removed list-wise (participant removed from analysis); missing data in facet scores were imputed with the facet mean, or removed list-wise. Multivariate tests for normality confirmed most variables were acceptable; positive skew was found in social facets. Squared transformations did not improve normality, so original values were analysed.

MANCOVA was used to test differences by group (symptom/lifestyle), using QoL domains as dependent variables (ANCOVA for general QoL). Dummy variables were calculated as needed, for socio-demographic and health covariates: age, gender, educational level, marital status, ethnicity, employment status, self-reported health status, and co-morbidities. Those with significant associations with the outcome variable were included in final M(ANCOVA)s. Where domains showed significant group differences, supplementary analyses of the domain’s component facets examined the details, and Bonferroni corrections adjusted for multiple comparisons (*p* = .006) [27].

A two-stage hierarchical multiple regression analysis was conducted to examine whether QoL domains predicted strength of help-seeking intention. Covariates established through MANCOVA were entered into Step 1 of the model; the four QoL domains entered at Step 2. A similar ANCOVA model examined general QoL.

## Results

Roadshow staff introduced 215 visitors to the researcher; five did not meet the inclusion criteria. The most frequent reason for declining was time constraints. A total of 109 enrolled in the study, of which two withdrew, giving a final sample of 107; one withdrew after starting because of time pressure, and one requested their data be withdrawn after participation.

### Descriptive statistics

Only 0.4% of total data were missing. Table 1 shows that there were equal numbers of men and women (mean age 53), and the majority were ethnically white (96%). More than half (57%) had completed only primary or secondary education; 66% were not employed. Similar proportions were single (42%) and married (39%). Forty-five percent were ill when tested (96% of this subsample reported a chronic illness and 2% an acute condition). The mean frequency of self-reported chronic co-morbidities was 0.5; most common was diabetes, chronic obstructive pulmonary disease, hypertension, and depression. Symptom (*n* = 61) and lifestyle (*n* = 46) groups were compared across all socio-demographic and health variables, using independent *t* tests and Chi-square.

**Table 1:** Demographic information for a community sample visiting the Cancer Research UK Roadshow

	Lifestyle group (*N* = 61)	Symptoms group (*N* = 46)	Total (*N* = 107)	*t*/*χ*^2^ (df)	*p*
Gender				.09 (1)	.759
Male	30 (49%)	24 (52%)	54 (50%)		
Female	31 (51%)	22 (48%)	53 (50%)		
Age				2.46 (105)	.016*
M (Range)	56 (18–90)	49 (18–80)	53 (18–90)		
Ethnicity				.55 (1)	.459
White	58 (95%)	45 (98%)	103 (96%)		
Not White	3 (5%)	1 (2%)	4 (4%)		
Highest education				1.20 (1)	.274
Primary/secondary	32 (52%)	29 (63%)	61 (57%)		
Tertiary	29 (48%)	17 (37%)	46 (43%)		
Occupation				.04 (1)	.844
In paid work	21 (34%)	15 (33%)	36 (34%)		
Not in paid work	40 (66%)	31 (67%)	71 (66%)		
Marital status				2.02 (2)	.365
Single	23 (38%)	22 (48%)	45 (42%)		
Married	24 (39%)	18 (39%)	42 (39%)		
Divorced/widowed	14 (23%)	6 (13%)	20 (19%)		
Health				2.94 (1)	.087
Currently ill	23 (38%)	25 (54%)	48 (45%)		
Not currently ill	38 (62%)	21 (46%)	59 (55%)		
Depression (PHQ-2)				− 3.66 (102)	.001**
*N* ≥ 3	9 (15%)	19 (41%)	28 (26%)		
M (SD)	1.24 (1.56)	2.40 (1.67)	1.74 (1.70)		

*p < .05, **p < .001

Results showed that the symptoms group was younger, *t*_(105)_ = 2.46, *p* < .05, and reported more depressive symptoms (PHQ-2), *t*_(102)_ = − 3.66, *p* < .001; no other significant group differences were found. Participants in the symptoms group reported an average of 1.33 symptoms/signs each. Of the 46 who reported a symptom/sign, 15% first noticed it in the last 2 weeks, 9% in the last month, 20% within 6 months, 17% the last year, 22% more than a year ago, and 17% three or more years ago. Table 2 shows the frequencies of the different symptom types reported by those in the symptom group.

**Table 2:** Frequencies of symptom types reported by those in the symptom group

Symptom type	Frequency	% of participants
Unexplained pain or ache	11	23.91
Sore that won’t heal	7	15.22
Unusual breast changes	5	10.87
Unusual lump	5	10.87
Changes to a mole	4	8.70
Change in bowel habit	4	8.70
Blood in faecal matter	4	8.70
Persistent cough	4	8.70
Problems urinating	3	6.52
Unexplained weight change	3	6.52
Breathlessness	2	4.35
Persistent heartburn or indigestion	2	4.35
Unexplained bleeding	2	4.35
Persistent bloating	1	2.17
Coughing up blood	1	2.17
Appetite loss	1	2.17
Mouth or tongue ulcer that won’t heal	1	2.17
Fatigue	1	2.17

### Comparison of QoL between symptom and lifestyle groups

When the QoL of the symptoms and lifestyle groups was compared (MANCOVA), no significant overall main effect was found for each QoL domain, after controlling for covariate effects, *V* = .081, *F*_(4,62)_ = 1.36, *p* = .258, depressive symptoms (PHQ-2:*V* = .435, *F*_(4,62)_ = 11.94, *p* < .001), and current illness (*V* = .248, *F*_(4,62)_ = 5.12, *p* < .01). However, univariate tests indicated a group difference in the psychological domain (*F*_(1,65)_ = 4.97, *p* < .05), due to lower QoL scores in the symptoms group (see Table 3), as predicted. An ANCOVA of group differences in general QoL also showed that depressive symptoms (PHQ-2 score), *F*_(1,66)_ = 13.80, *p* < .01, and current illness, *F*_(1,66)_ = 5.24, *p* < .05, were significant covariates. With covariates controlled, significant group differences were found for general QoL *F*_(1,66)_ = 4.80, *p* < .05, due to lower QoL in the symptoms group. Both psychological and general QoL results confirmed predictions that lower QoL would be reported by the symptoms group.

**Table 3:** Differences in QoL between symptom and lifestyle groups

WHOQOL domain (bold)/facet	Lifestyle group (*N* = 37)	Symptom group (*N* = 35)	F (df)	*p*
	M (SD)	M (SD)		
**Core QoL**				
**General QoL**	3.66 (.76)	3.01 (.87)	4.80 (1)	.032*
QoL	4.00 (.67)	3.29 (.96)	7.10 (1)	.009
Health	3.32 (1.05)	2.74 (.98)	1.13 (1)	.266
**Physical**	66.97 (20.41)	50.61 (20.98)	3.23 (1)	.077
**Psychological**	67.23 (16.08)	50.24 (21.12)	4.97 (1)	.029*
Positive feelings	3.76 (.92)	3.14 (1.03)	1.50 (1)	.226
Thinking, learning, memory and concentration	3.65 (.79)	3.26 (1.09)	1.80 (1)	1.84
Self-esteem	3.70 (1.00)	2.69 (1.02)	8.19 (1)	.006**
Body image and appearance	3.54 (1.10)	3.03 (1.40)	.09 (1)	.770
Negative feelings	3.54 (1.04)	2.80 (1.23)	2.32 (1)	.132
Spirituality, religion and personal beliefs	3.84 (.80)	3.20 (1.21)	2.55 (1)	.115
**Social**	64.86 (23.74)	54.66 (21.44)	1.14 (1)	.290
**Environmental**	70.86 (15.40)	60.27 (17.90)	1.89 (1)	.174

*p < .05, **p = .006 (Bonferroni correction)

Poorer QoL in specific facets could account for group differences in significant domains. As this information has potential pragmatic value, assisting clinicians to pinpoint and act upon particular QoL facets, we conducted MANCOVA tests for group differences in facets of the psychological domain and general QoL to explore this detail. Covariates included were employment status, illness status, and depressive symptoms, and Bonferroni adjustments were made for multiple comparisons (see Table 3). Only self-esteem (psychological domain) was significantly poorer in the symptoms group, *F*_(1,66)_ = 8.19, *p* = .006.

### Intention to help-seek

A two-stage hierarchical multiple regression analysis examined the relationship between the strength of intention to seek help and QoL (see Table 4). Age and group (symptom/lifestyle) were established as covariates. It was expected that presence of a current illness would be related to stronger intention to visit the GP, but this was not confirmed (*F*_(1,100)_ = 1.82, *p* = .18). Covariates were entered at Step 1, and the four QoL domain scores at Step 2.

**Table 4:** Regression showing relationship between QoL domains and intention to help-seek

	B	SE B	β
Step 1			
Constant	− 1.95	.89	
Age	− .01	.01	− .08
Group	1.65	.34	.51**
Step 2			
Constant	− 1.14	1.11	
Age	− .01	.01	− .07
Group	1.26	.37	.39*
Physical	− .03	.01	− .37*
Psychological	− .01	.01	− .17
Social	.01	.01	.13
Environmental	.02	.01	.25

*R*^2^ = .28 for Step 1, Δ*R*^2^ = .11 for Step 2 (*p* < .05), *p < .05, **p < .001

The model was significant overall, *F*_(6,61)_ = 6.28, *p* < .001, with adjusted *R*^2^ = .32, explaining 32% of the total variance in help-seeking. The predictors showed that level of physical QoL significantly predicted the strength of intention to seek help from a GP in the *next* 2 weeks, as the beta-value showed that lower physical QoL predicted stronger intentions to consult. In a similar general QoL model, no significant effects were found.

## Conclusions

This preliminary study examined the QoL of people in the community with a potential symptom/sign of cancer. Being able to assess their QoL *before* they consulted their GP offers an original contribution, as little is known about how this population responds during the earliest stage of the Pathway to Treatment [6]. Our findings confirmed poorer QoL for those with potential cancer symptoms during the period immediately prior to visiting the roadshow, in comparison to a group seeking advice about lifestyle changes to reduce cancer risks. Not only was overall QoL and health lower for those with symptoms/signs, but lower psychological QoL scores also showed poorer mental health. These findings are consistent with previous research suggesting that the presence of physical symptoms can reduce QoL [8, 9]. It is interesting to note that there was no significant difference in the physical domain of QoL; however, inspection of mean QoL in each group shows that QoL was consistently lower in the symptoms group across all domains, by a considerable margin. It is possible that these differences did not reach significance due to the small sample size and should be reinvestigated further in a larger sample. Diagnosed cancer patients report that cancer negatively affects their QoL [10, 28, 29], but the present study provides the first preliminary evidence that QoL deterioration begins much earlier in the pathway than previously recorded and is present before they initially consult their GP.

Finally, this study demonstrates that lower physical QoL was associated with stronger intentions to consult a GP in the 2 weeks following the roadshow visit, even after controlling for the presence of a potential cancer symptom, and thus whether roadshow staff had advised consulting a GP. Therefore, it is not merely the presence of a symptom that promotes intention to seek help but rather the effect of the symptom on QoL. This finding supports the prediction that QoL levels appear to be a contributory factor motivating decisions concerning whether and when to seek help, among several other known factors. The present research adds to rare studies addressing this question at the time of the symptom experience, without exclusive reliance on retrospective indices of help-seeking [20, 21].

### Clinical implications

The study findings have implications for research and practice related to the ‘patient interval’ in cancer diagnosis, comprising of two periods; one of appraisal and one help-seeking [30]. The Pathways to Treatment model suggests that between detecting a bodily change and perceiving a reason to discuss it with an HCP, patients undergo a process of appraisal and self-management which is influenced by psychological, social and cultural factors [6]. These factors are integral to the WHOQOL definition of QoL and its assessment, pointing to suitability of the WHOQOL-BREF assessment for quantifying them. Both general QoL and psychological QoL differed between lifestyle and symptom groups. Although research with larger samples and supplementary methods will be necessary to better establish such differences, this study highlights generic QoL assessment as a promising avenue for research. Evidence that poorer physical QoL is associated with stronger help-seeking intentions suggests that physical QoL levels represent a motivating factor, driving help-seeking behaviour. While further longitudinal work will be necessary to rule out reverse causality in the relationship between QoL and intention to seek help, the establishment of their association here highlights their likely intertwined roles during the patient interval. An implication is that interventions to increase awareness of changes in physical QoL concurrent with a bodily change could promote earlier help-seeking behaviours, and ultimately aid earlier cancer diagnosis. Interventions could highlight connections between emerging symptoms/signs, and associated reductions in physical QoL, using media channels (e.g., online videos), primary care wellbeing clinics, charity (Cancer Research UK) websites, community clinics (e.g., contraceptive; smoking cessation), and education (e.g., school wellbeing classes). These findings therefore have economic implications for more efficient delivery of healthcare to those in most need.

The length of time that some participants had lived with a symptom without reporting it was notably long, with 17% reporting that their symptoms started three or more years ago. This represents a significant problem for oncology, as delays in the diagnosis and treatment of particular types of cancer can lead to a reduced chance of survival [5]. Furthermore, many of the studies which report the length of the patient interval rely on retrospective accounts of people who have gone on to be diagnosed later. For example, Ristvedt and Trinkaus [31] reported a mean delay time of about 6 months for people with symptoms of rectal cancer, with around 17% waiting a year or more. It is not specific to oncology that the scale of the problem is far greater than suggested by looking at research samples such as ours. Our findings confirm that there are people who live with potentially serious symptoms for years without reporting them, and indeed who may never report them (e.g., [32]). It is imperative that work continues to ensure that people report potentially serious symptoms promptly, and to reduce the time between detection and consultation.

Previous research demonstrated the feasibility of collecting subjective QoL information from ill and well people in non-deprived rural and urban communities [33]. However, the present study extends this work to areas of high socio-economic deprivation, as reflected by the sample characteristics: 57% only completed primary or secondary education, compared with the UK average of 37%, and 66% were not in paid work, compared to the national average of 39% [34, 35]. These features illustrate some of the challenges of investigating QoL and reporting behaviour in a ‘hard-to-reach’ population. Despite the sample limitations, these preliminary findings are rare and therefore valuable. Although the generalisability of the findings is limited, early diagnosis among deprived populations is particularly important because lower socioeconomic groups are disproportionately affected by premature cancer deaths [36, 37].

Although not the prime study focus, we recruited an unusually high proportion of men (50%) to this health study. Men are generally less likely than women to seek a GP consultation and a recent epidemiological study shows that men, and socio-economically disadvantaged adults, are more likely to be diagnosed with cancer in hospital emergency departments, having not seen their GP beforehand [38, 39]. As the roadshow appears to lower barriers to seeking advice among men, this community health service appears to actively contribute to redressing gender inequalities in health. Moreover, this facilitative effect persisted when men were approached about participating in the present research. Although small scale, these findings suggest that men are more likely to seek an unplanned, spontaneous consultation than make and attend a planned GP appointment. Such findings have particular attendance and cost implications for the NHS.

### Study limitations

Recruiting those experiencing a potential cancer symptom before consulting their GP was a major challenge, resulting in a smaller sample size than planned. Cancer Research UK Roadshow visitors that fit the inclusion criteria were fewer than estimated, and refusals were mainly because of time shortage. In addition, this was a self-selecting, not a random sample. Nevertheless, the rationale for studying this population remains strong, as it is essential to understand decision-making processes underlying help-seeking behaviour within socio-economically deprived communities. Despite recruitment difficulties, we make the following recommendations for future studies. Public engagement events focussed on cancer are valuable, particularly when community-based, and attractive to passers-by. Creating a friendly, inviting and interactive interface is essential and can be achieved with signage and health-relevant props. With low recruitment at individual sites, attendance of multiple recruiters at many sites and events is recommended. Furthermore, direct recruitment of participants by the research team may improve enrolment rates. As time restrictions were the primary reason given for non-participation, studies should minimise the amount of time required to participate. Cancer Research UK roadshow locations are chosen for prominence in high-footfall areas but offer private space to promote discussion of potentially sensitive issues with qualified nurses; this may not occur in public settings. Recruitment for the current study could have been impeded by the fact that data was collected in a tent; exposure to weather and lack of complete privacy may have deterred some participants. Future research should endeavour to provide the most comfortable and private environment possible.

In conclusion, this unique study makes a distinctive contribution to understanding the QoL of people experiencing a potential cancer symptom/sign before they consult their GP. There was preliminary evidence for reduced QoL in this sample compared to those considering lifestyle changes. In addition, poorer physical QoL predicted stronger intention to consult the GP, representing an important motivational factor in the decision to consult primary care. Changes in QoL during this pre-consultation period are an important area for future research. Interventions designed to increase QoL awareness could combine with other known factors to promote timely help-seeking for cancer symptoms.
